# Residual Dynamics of Chlorantraniliprole and Fludioxonil in Soil and Their Effects on the Microbiome

**DOI:** 10.3390/jox15010004

**Published:** 2024-12-30

**Authors:** Nan Hao, Huimin Zhang, Hui Jia, Yuwei Zhao, Jiaqi Li, Xiaoxiao Feng, Bowen Tang, Bin Zhao, Yingchao Liu

**Affiliations:** State Key Laboratory of North China Crop Improvement and Regulation, College of Plant Protection, Hebei Agricultural University, Baoding 071001, China; h15832215606@163.com (N.H.); ndhminzhang@126.com (H.Z.); hui_jiahui@126.com (H.J.); z18715912135@163.com (Y.Z.); ljq1819366@163.com (J.L.); fengxiaoxiao@hebau.edu.cn (X.F.); zhbtbw@hebau.edu.cn (B.T.)

**Keywords:** chlorantraniliprole, fludioxonil, residual digestion dynamics, microbiome

## Abstract

The increased use of chlorantraniliprole and fludioxonil has sparked concerns about their residues and impact on the soil microbiome, highlighting an urgent issue requiring attention. This study investigates the residue dynamics of corn after chlorantraniliprole and fludioxonil treatments, as well as their effects on soil enzyme activity and microbial community structure. High-performance liquid chromatography–tandem mass spectrometry (HPLC-MS/MS) analysis showed a significant decrease in chlorantraniliprole and fludioxonil residues in the soil after combined application, especially with chlorantraniliprole. This application caused a temporary reduction in urease and sucrase activities. Furthermore, high-throughput sequencing of the soil microbiome revealed a decrease in the relative abundance of *Talaromyces* during fludioxonil application, while *Mortierela* and *Gibberella* increased. Additionally, *Vicianmibacteraceae* and *Vicianminbactererales* saw significant increases after chlorantraniliprole application. The combined application of chlorantraniliprole and fludioxonil not only decreased the population of harmful microorganisms but also lowered residue levels in the soil when compared to individual applications. This ultimately enhanced the efficacy of control measures and promoted environmental compatibility.

## 1. Introduction

*Zea mays* L. is a key food crop in China. Changes in agricultural practices, such as no-till adoption, straw return, and crop rotation, have led to a rise in pest and disease prevalence and damage in maize fields in recent years [1]. To address these challenges and improve corn quality, the use of pesticides has become essential. There is currently a trend towards using a combination of different pesticides to prevent and control corn diseases and insect pests. Chlorantraniliprole (CAP), an anthranilic diamide insecticide, is effective against various pests and is considered a low-risk pesticide due to its minimal impact on mammals and non-target arthropods [2]. On the other hand, fludioxonil (FLU) is a novel phenylpyrrole fungicide that inhibits fungal hyphae growth by disrupting glucose phosphorylation transport, ultimately leading to the demise of pathogenic bacteria. Known for its low toxicity, prolonged efficacy, and minimal dosage requirements, FLU is well suited for various applications [3]. The combined use of CAP and FLU as a seed treatment agent, as indicated by patents, can broaden the spectrum of bactericidal activity and effectively prevent various crop seedling diseases [4].

The environmental fate of pesticides post-application has been extensively studied. The increasing use of CAP and FLU has drawn global attention to the presence of their residues in crops and soil. Studies have indicated that the residual levels of FLU in brown rice and rice stalks are below 0.002 mg/kg after 154 days of using 20% FLU WP as a seed dressing agent, meeting the maximum residue limit [5]. Research has also explored the degradation of CAP in NRRI alluvial soil and red paddy soils; the initial residues were found to be 0.037 μg/g and 10.043 μg/g, with the dissipation rate of CAP in both soils exceeding 89% after 60 days of treatment [6]. However, there is a gap in knowledge regarding the degradation pattern of residues when both pesticides are combined in corn field soil, necessitating further investigation in this specific context. Pesticides play a vital role in food production but can have detrimental effects on the environment and ecosystems. When applied to seeds, pesticides may be released into the air during planting, potentially harming above-ground insects [7]. Nevertheless, most chemicals in seed coatings target the rhizosphere, the soil area where seedling roots develop. While effective against certain soil-borne pathogens and herbivores, these pesticides lack specificity for particular species [8]. Unfortunately, our understanding of their impact on non-target microorganism communities is limited. Additional research is needed to evaluate the ecological repercussions of seed-applied pesticides on non-target microbiomes and the overall soil ecosystem, which is crucial for the advancement of sustainable and environmentally friendly agricultural practices.

Current research on CAP and FLU primarily focuses on the behavior of these compounds in soil and has mainly looked at their persistence and dissipation in specific cropping systems. However, there is a lack of information regarding the impact of CAP and FLU on soil microbial activity and community composition. To address this knowledge gap, this study conducted indoor pot experiments to explore the effects of CAP and FLU on soil enzyme activities (urease, invertase, and dehydrogenase), soil microbial abundance, and soil microbial community structure. The main objective was to gain a deeper understanding of how these compounds influence soil microbial communities and enzymatic activity in the rhizosphere soil of maize. The findings of this study will offer valuable insights for evaluating the potential environmental risks associated with CAP and FLU.

## 2. Materials and Methods

### 2.1. Experimental Soil Source and Experimental Design

Soil samples were obtained from the cultivated layer (0–15 cm) of the greenhouse experimental field at the West Campus of Hebei Agricultural University in Baoding, China. The soil was classified as alkaline, with a pH of 8.2 and organic matter content of 20.06 g/kg. Notably, the field had not been treated with CAP or FLU for 5 years. Prior to the experiment, the soil was air-dried and sieved through a 2 mm mesh for later use. The seed coating agents were 50% CAP (DuPont, WLM, DE, USA) and 2.5% FLU (Syngenta Nantong Crop Protection, Nantong, China). The seed treatments included four different coating treatments, CK (untreated seeds), T1 (5.3 g of 50% CAP in 1 kg of corn seeds), T2 (2 mL of 2.5% FLU in 1 kg of corn seeds), and T3 (mixed T1 and T2 with a ratio of 1:1). Residue detection and enzyme activity were analyzed by adding 5 corn seeds to each 250 g soil sample, which were retrieved when treated for 2 h, 3 d, 7 d, 14 d, 28 d, 42 d, 60 d, and 90 d. Additionally, rhizosphere soil samples of corn were collected at 0 d, 7 d, 35 d, 60 d, and 90 d, respectively, by excavating the whole plant to isolate soil adhering to the roots. All experiments were performed in three biological replicates.

### 2.2. CAP and FLU Analysis by HPLC-MS

The analytical method for CAP and FLU was validated following the laboratory validation protocol. HPLC analysis was performed using a Bonshell C-18 Column (50 mm × 2.1 mm × 2.7 μm), with a flow rate of 0.3 mL/min, column incubator set at 40 °C, injection volume of 2 μL, sample chamber temperature at 15 °C, and mobile phase consisting of 0.1% formic acid in water and acetonitrile. Mass spectrometry conditions for FLU included a precursor ion of 247, with product ions at 180 and 126, while CAP had a precursor ion of 484 and product ions at 285.9 and 253. The mass concentrations of CAP and FLU ranged from 0.005 to 0.5 mg·kg^−1^, with linear equations y = 995.8x + 2.073 (R^2^ = 0.9987) for CAP and y = 915.1x + 3.086 (R^2^ = 0.9982) for FLU. The mean addition recoveries of CAP were between 100 and 113% with relative standard deviations (RSDs) ranging from 1% to 7%, while for FLU, they ranged from 96% to 104% with RSDs from 2% to 6%. Both met the requirements for pesticide residue detection. The sample processing method was as follows: Initially, 5.0 g of soil was measured and mixed with 100 mL of deionized water for 30 min. Subsequently, 20 mL of acetonitrile and 5.0 g of NaCl were added. The resulting supernatant, obtained after centrifugation, was filtered through a 0.22 µm organic filter membrane before being transferred to an autosampler vial for HPLC-MS analysis.

### 2.3. Determination of Soil Enzyme Activities

To assess the ecological toxicity of CAP and FLU, the activities of urease, sucrase, and dehydrogenase were determined using the methods referenced in previous studies. A total of 1 mL of toluene was added to 5 g of soil and shaken for 15 min. Then, 5 mL of 10% urea solution and 10 mL of citrate buffer solution (pH = 6.7) were added. The mixture was left at 37 °C for 24 h, diluted to 50 mL with distilled water, and filtered. From the filtrate, 1 mL was combined with 10 mL of distilled water, 4 mL of sodium benzoate solution, and 3 mL of sodium hypochlorite solution for 20 min to measure absorbance at 578 nm for urease enzyme activity. For sucrase enzyme activity, 15 mL of 8% sucrose solution, 5 mL of pH 5.5 phosphate buffer, and 5 drops of toluene were added to 5 g of soil, incubated at 37 °C for 24 h, filtered, and then tested using a DNS solution to determine activity.

### 2.4. Determination of Soil Microbial Counts

Soil microbial colonies were counted using the spread plate method. Initially, 10 g of soil was mixed with 90 milliliters of sterile water, shaken, and left to settle to create a 10^−1^ soil microbial suspension. This suspension was then diluted with sterile water to create 10^−2^, 10^−3^, 10^−4^, and 10^−5^ soil microbial suspensions. Fungi were incubated at 28 °C for 5 days, while bacterial and actinomycete samples were cultured at 37 °C for 1 and 5 days, respectively. The experiment was performed in triplicate. The number of colonies per gram of soil was calculated by dividing the average number of colonies by the quantity of soil used and multiplying by the dilution factor.

### 2.5. Soil DNA Extraction and Illumina MiSeq Sequencing

DNA extraction was performed per test pot; each treatment had 3 replicates, and the control soil was the soil without the application of the agent. Microbial community genomic DNA was extracted from soil samples using the E.Z.N.A soil DNA Kit (Omega Bio-tek, Norcross, GA, USA) following the manufacturer’s instructions. The 16S rRNA gene was amplified with primer pairs 338F and 806R, while the ITS1 and ITS2 genes were PCR-amplified using fungal universal primers. Quantification of purified DNA was performed with the Quantus™ Fluorometer (Promega, Madison, WI, USA). Library construction utilized the NEXTflexTM Rapid DNA-Seq Kit (San Diego, CA, USA), and sequencing was conducted on the Illumina Miseq PE300/NovaSeq PE250 platform (Shanghai, China).

### 2.6. Processing of Sequencing Data

The raw 16S rRNA gene sequencing reads underwent demultiplexing and quality filtering with fastp version 0.20.0. Subsequently, the filtered reads were merged using FLASH version 1.2.7. Operational taxonomic units (OTUs) were clustered at a 97% similarity cutoff utilizing UPARSE version 7.1. Chimeric sequences were identified and eliminated from the dataset. The taxonomic classification of each OTU representative sequence was conducted using the RDP Classifier version 2.2 against the 16S rRNA database (version 138) with a confidence threshold of 0.7.

The α diversity index, calculated in Majorbio, is used to assess microbial diversity in the soils. Microbial community composition variations are depicted through principal coordinate analysis (PCoA) based on the Bray–Curtis distance. In microbial network analysis, co-occurrence networks are examined at the OTU level, with a Pearson correlation coefficient higher than 0.8 and a *p*-value lower than 0.01 indicating statistical reliability. The Gephi interactive platform is employed for visualizing the network using Spearman visualization [9].

### 2.7. Data Analysis

Residues of CAP and FLU in soil were detected using HPLC-MS/MS. The mean and standard error (SE) were calculated from triplicate repeated measurements and then plotted using OriginLab 2021.

Soil enzyme activity and soil microbial population at different time points were detected using both the BioTek MicroplateReader and the spread plate method. Each time point had three replicates. The mean and standard error (SE) were calculated from the data obtained from these replicates. ANOVA combined with Duncan’s test was used to determine the significance of the differences between the samples at each time point; different letters indicate significant differences (*p* < 0.05). Statistical analysis was performed using SPSS 26, and the figures were created using OriginLab 2021.

The biodiversity of organisms was analyzed based on the Shannon and Simpson indices of α diversity. The mean and standard error (SE) were calculated from three repeated measurements and plotted using Origin 2021. Visualization of the microbial community composition differences was based on principal coordinate analysis (PCoA) using the Bray–Curtis distance. The community composition analysis and fungal function prediction were conducted using the Bar plot, Pie plot, and FUNGuild functions on the Majorbio website, respectively, and the grouped samples were calculated as the median. In the microbial network analysis, the co-occurrence network was examined at the OTU level; the Pearson correlation coefficient was higher than 0.8, and the *p*-value below 0.01 indicated statistical reliability, which was used with Spearman’s correlation to visualize the network using the Gephi interactive platform.

## 3. Results

### 3.1. Residual Resolution Dynamics of CAP and FLU in Soil

**Figure 1:**
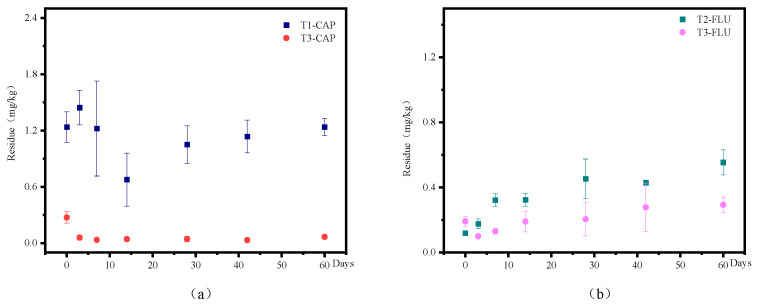
Degradation dynamics of CAP (**a**) and FLU (**b**) in soil at different periods. Note: Residues of CAP and FLU in maize soil at different times were detected by HPLC-MS/MS with three replicates at each time point, and the error bars represented the standard error (SE), which was calculated and graphed using Origin 2021.

The residual concentrations of CAP and FLU in soil samples were determined using an HPLC-MS/MS method. Regression analysis revealed a strong linear relationship between the mass concentration of CAP and FLU and their respective peak areas within the concentration range of 0.005–0.5 mg/L. The linear regression equations obtained were y = 3.97x + 0.0089 (R^2^ = 0.998) for CAP and y = 3.976x + 0.0093 (R^2^ = 0.998) for FLU. Figure 1 depicts the degradation dynamics of CAP and FLU in soil, showing their residues post-seed germination. When CAP was used alone, its residue initially increased and then decreased, whereas FLU alone exhibited an initial rise followed by stabilization. When used in combination, the residues of CAP and FLU were lower compared to individual applications, particularly for CAP. In conclusion, the degradation dynamics of CAP and FLU in soil were influenced by their individual and combined applications, with CAP dissipating more rapidly in soil when combined with FLU.

### 3.2. Soil Enzyme Activities of CAP and FLU

**Figure 2:**
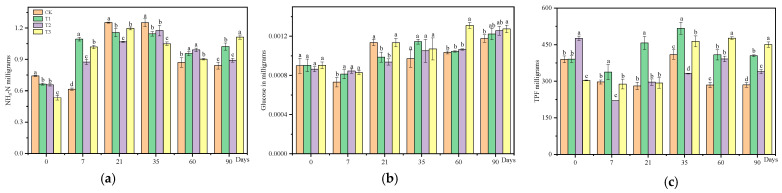
The study investigated the impact of CAP and FLU exposure on soil enzyme activities. Each bar in the graph represents the mean of three replicates, with error bars indicating the standard deviation. Different letters above the columns signify significant differences at a *p*-value of less than 0.05 between treatments and control. (**a**) Effect of agent on urease. (**b**) Effect of agent on sucrase. (**c**) Effect of agent on dehydrogenase. Note: The absorbance changes in urease, sucrase, and dehydrogenase after application of CAP and FLU at different times were determined using a microplate reader according to the detection method in Section 2.3, with three replicates at each time point, and the error bars represented the standard error (SE). ANOVA combined with Duncan’s test was used to determine the significance of the differences between the samples at each time point; different letters indicate significant differences (*p* < 0.05).

The soil enzyme responses to various treatments of CAP and FLU are depicted in Figure 2. Urease activity gradually increased across all treatments from 0 d to 7 d compared to the control group. Subsequently, between 7 d and 35 d, urease activity in the treatment groups notably decreased below that of the control. However, it gradually recovered in all treatment groups, and by the end of the experiment, it returned to control levels. Both T1 and T3 treatment groups exhibited significantly higher urease activity compared to the control group. After the administration of the agent, a transient stimulatory effect was noticed on sucrase activity. However, both the T1 and T2 treatments exhibited an initial decrease after 7 days, followed by a gradual recovery by 35 days, surpassing the levels observed in the control group. Dehydrogenase activity in the T1 and T3 treatment groups consistently remained higher than that of the control group throughout the 90-day experiment. In addition, at 7 d and 35 d post-experiment, dehydrogenase activity in the T2 treatment group was lower than the control. However, for the remainder of the experiment, dehydrogenase activity in the T2 treatment group was significantly higher than the control, with T1 and T3 treatments also enhancing dehydrogenase activity. In summary, different treatments of CAP and FLU initially enhanced and then inhibited urease and sucrase activities, followed by a recovery phase surpassing the control level. Dehydrogenase activity, on the other hand, was consistently increased by T1 and T3 treatments throughout the experiment, while T2 treatment briefly inhibited dehydrogenase activity before rebounding to surpass control group levels.

### 3.3. The Impact of CAP and FLU Treatments on the Number of Microorganisms

**Figure 3:**
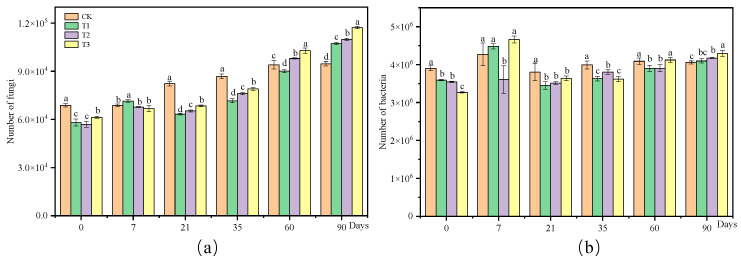
The impact of CAP and FLU on the number of microorganisms. (**a**) The total number of fungal communities. (**b**) The total number of bacterial communities. Note: The change in the number of microorganisms after application of CAP and FLU at different times was determined by the spread plate method according to the detection method in Section 2.4, with three replicates at each time point. Error bars represent standard errors. ANOVA combined with Duncan’s test was used to determine the significance of the differences between the samples at each time point; different letters indicate significant differences (*p* < 0.05).

The variations in the number of soil microorganisms under different treatments were analyzed. In Figure 3a, fungal colonies in the T1 treatment surpassed those in the control group on day 7, while no significant difference was noted between the T2 and T3 treatments compared to the control. Subsequently, between 7 d and 35 d, fungal colonies in all treatments notably decreased in comparison to the control. However, post-35 days, fungal colonies in each treatment group showed an increase and eventually reached control levels. At the end of the experiment, the number of colonies in T1, T2, and T3 treatments was significantly higher than in the control group. Regarding bacteria (Figure 3b), on day 7, the number of bacterial colonies was lower than the control group, except for the T2 treatment group. From day 7 to day 35, the number of bacterial colonies in all treatment groups remained below that of the control group. After 35 d, the number of bacterial colonies in each treatment group gradually increased, ultimately returning to control levels. At the conclusion of the experiment, the number of bacterial colonies in T2 and T3 treatments exceeded that of the control group.

### 3.4. Impact of CAP and FLU on Soil Microbial Communities in Corn Rhizosphere

**Figure 4:**
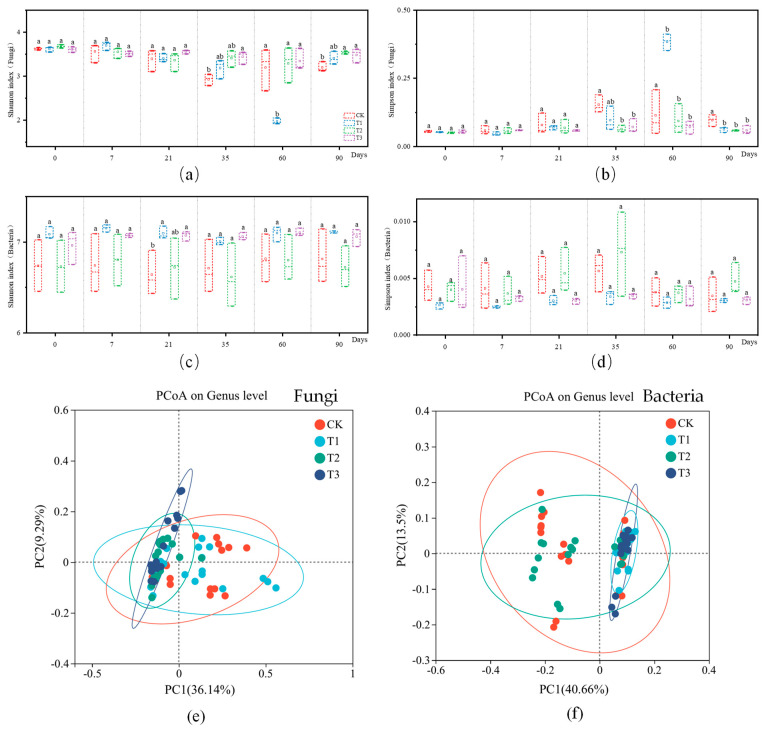
Alpha diversity indicators (Shannon index and Simpson index) and PCoA analysis were used to compare changes in fungal and bacterial diversity over time under different soil treatments. (**a**) Fungal diversity analysis (Shannon index). (**b**) Fungal diversity analysis (Simpson index). (**c**) Bacterial diversity analysis (Shannon index). (**d**) Bacterial diversity analysis (Simpson index). (**e**) Fungal PCoA analysis. (**f**) Bacterial PCoA analysis. Note: (**a**–**d**): Biological diversity analysis was based on Shannon and Simpson metrics using high-throughput sequencing results obtained in 2.5, with three replicates for each time point, graphically plotted using Origin 2021. (**e**–**f**): Characterization of microbial community composition changes was based on principal component analysis (PCoA) using Bray–Curtis distance and the results of high-throughput sequencing performed in 2.5.

Amplicon sequencing was performed on soil samples to assess the effects of CAP and FLU treatments on fungal and bacterial communities in corn rhizosphere soil. Soil bacterial and fungal diversity were measured using the Shannon and Simpson indices after exposure to CAP, FLU, and a combination of both. Overall, biodiversity showed a positive association with the Shannon index and a negative relationship with the Simpson index. Fungal diversity, as indicated by the Shannon and Simpson indices, consistently increased over time, suggesting that all three treatments contributed to enhancing fungal diversity (Figure 4a,b). Conversely, bacterial diversity exhibited short-term fluctuations but eventually returned to baseline levels after 35 d, indicating that the treatments did not have a lasting impact on bacterial diversity (Figure 4c,d). These results imply that microbial diversity remained relatively stable in the long term following exposure to CAP, FLU, and their combination. The impact of CAP and FLU on soil microorganism community structure at the genus level was assessed using principal coordinate analysis (PCoA) for both fungal and bacterial communities across different treatment groups. The findings showed significant differences in fungal community structure between the control group and the T2 and T3 treatment groups. However, there was no significant separation observed in the bacterial community between the T2 and T3 treatment groups (Figure 4e,f). FLU application notably influenced the fungal community structure, while CAP had a significant effect on the bacterial community structure. In conclusion, CAP and FLU had distinct impacts on bacterial and fungal communities, respectively.

### 3.5. Impact of CAP and FLU on the Composition of Soil Microbial Community in Corn Rhizosphere

**Figure 5:**
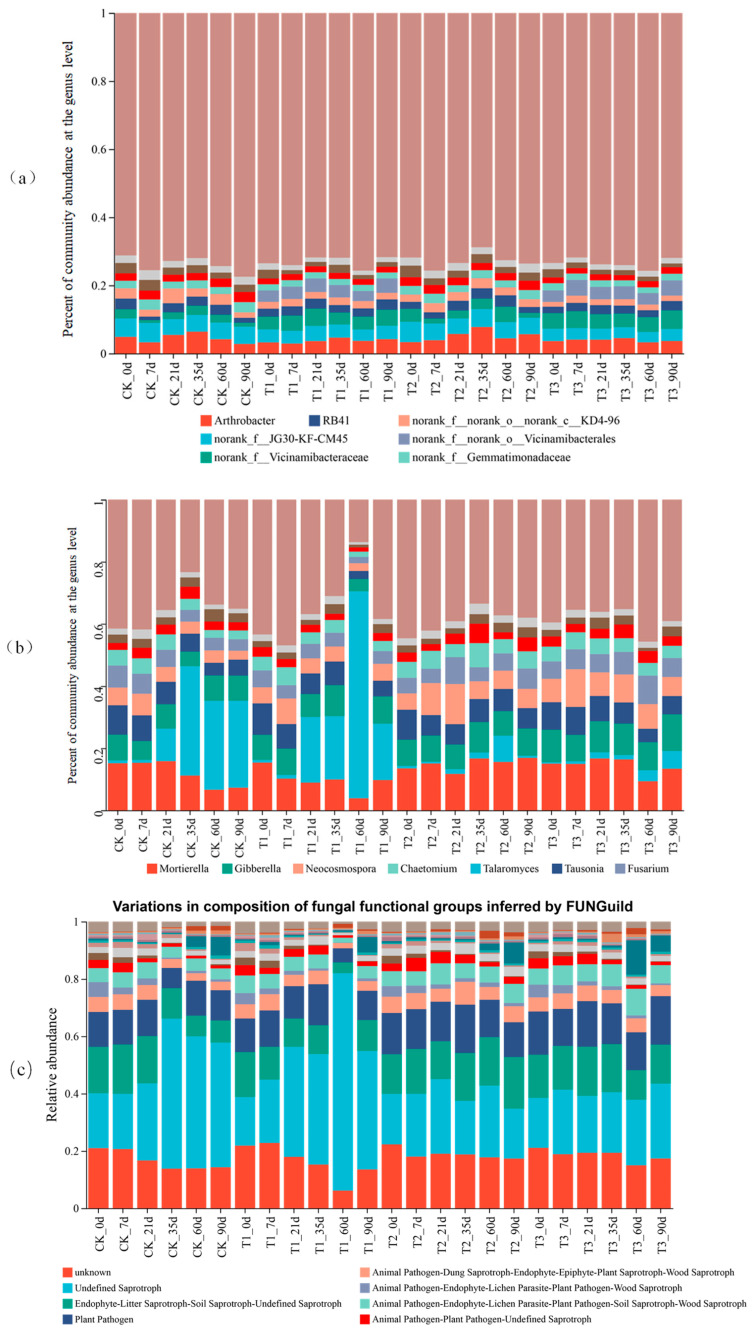
Soil microbial communities and the microbial functional diversity in soil. (**a**) Fungal community structure analysis. (**b**) Bacterial community structure analysis. (**c**) FUNGuild function prediction of endophytic fungi. Note: (**a**,**b**): The community composition was analyzed using the Bar plot and Pie plot of the community on the Majorbio website; the taxonomic level was genus, the grouped samples were calculated as the median, and the top 10 abundance rankings were selected for graphing. (**c**): The functional classification of fungi and the abundance information of each functional class in different samples were analyzed using the FUNGuild functional prediction in the Majorbio website, and the grouped samples were calculated as the median.

The study identified *Mortierella*, *Talaromyces*, *Gibberella*, *Tausonia*, *Neocosmospora*, *Fusarium*, and *Chaetomium* as the top seven dominant genera in terms of relative abundance in the fungal community. *Mortierella*, *Talaromyces*, and *Gibberella* were highlighted as the primary fungal communities, with *Gyrothrix* and *Waitea* also present at lower abundance levels. The application of CAP and FLU influenced the relative abundance of these fungal genera, leading to notable shifts in *Mortierella*, *Talaromyces*, and *Gibberella* following the use of FLU alone or in combination with CAP. Specifically, *Mortierella* and *Gibberella* decreased, while *Talaromyces* also exhibited a reduction (Figure 5a and Figure S1a). To explore the impact of these fungicides on the functional composition of soil fungal communities in the rhizosphere, FUNGuild was utilized to predict functional groups. The analysis revealed various functional groups, including undefined saprotrophs, endophyte-litter saprotroph-soil saprotroph-undefined saprotrophs, and plant pathogens. A comparison with the control group showed that certain functional groups, such as undefined saprotrophs, decreased with the use of FLU, while endophyte-litter saprotroph-soil saprotroph-undefined saprotrophs increased (Figure 5c and Figure S1b). The distribution of bacterial communities at the genus level was analyzed using 16S rRNA amplicon sequencing. Significant genera identified included JG30-KF-CM45, *Arthrobacter*, *Vicianmibacteraceae*, *Vicianmibacterales*, RB41, KD4-96, *Gemmatimonadaceae*, and other bacterial taxa. The results indicate that the presence of CAP and FLU affects the soil bacterial community. Specifically, the use of CAP, either alone or in combination with FLU, led to a notable increase in the abundance of *Vicianmibacteraceae* and *Vicianmibacterales* (Figure 5b and Figure S1c). However, FLU alone did not cause a significant shift in bacterial abundance compared to the control group.

### 3.6. Interactions between Bacteria and Fungi in Soil Microbial Communities under Pesticide Treatments

**Figure 6:**
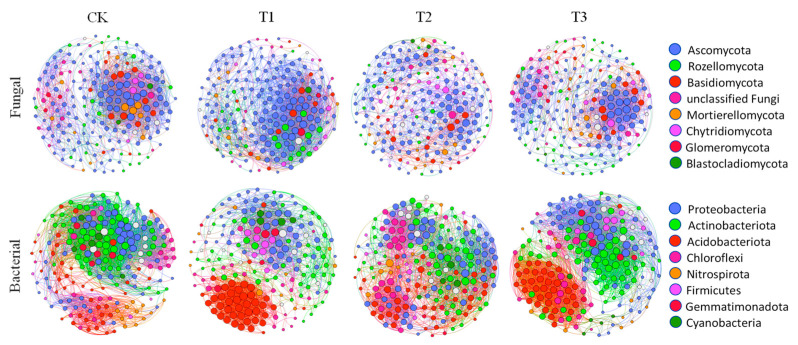
The ecological network among microbiomes. Note: In the microbial network analysis, the co-occurrence network was examined at the OTU level. The Pearson correlation coefficient was higher than 0.8, and the *p*-value below 0.01 indicated statistical reliability, which was used to visualize the network with Spearman’s correlation using the Gephi interactive platform.

By constructing a collinear network map of fungi and bacteria, we investigated the complex interactions within soil microbial communities under different pesticide treatments (Figure 6). Our results revealed distinct collinear networks for CAP, FLU, and the combination treatment compared to the control group. Among fungi, Figure 6 and Table S1 show that *Ascomycota* and *Mortierellomycota* are the dominant species in each treatment, and the nodes of *Mortierellomycota* after T2 and T3 treatments have an upward trend, which may be the symbiosis between *Ascomycota* and *Mortierellomycota*. In the bacterial collinear network analysis, JG30-KF-CM45 was identified as a member of the *Chloroflexi* phylum, while *Vicianmibacteraceae* and *Vicianmibacterales* were found in the *Acidobacteriota* phylum. As shown in Figure 6 and Table S2, the number of nodes of *Acidobacteriota* increased significantly after T1 and T3 treatments, dominating the T1 and T3 treatment communities, and according to the change in summary nodes, the number of nodes decreased after the application of CPA, indicating that the complexity of the network was reduced.

## 4. Discussion

The combination of CAP and FLU as a seed treatment can expand the range of bacteria they can inhibit, thereby preventing various seedling diseases in crops. However, there is a research gap regarding the impact of CAP and FLU residues in soil after using this mixture on microbiomes. The study aims to investigate the residues of CAP and FLU in soil after applying the mixture as a seed treatment and assess their effects on microbial abundance, enzyme activities, and microbial communities in the rhizosphere soil.

### 4.1. FLU Reduces the Residual Effectiveness of Its Compounded Agent

The research discovered that combining the two pesticides led to reduced residues compared to using them separately, with a notable decrease in CAP residues. Additionally, the study examined the degradation patterns and distribution of Metalaxyl-M and FLU in a 6.25% Metalaxyl-M·FLU Seed Coating Agent on soybean seeds, roots, leaves, and the surrounding soil. It was noted that FLU persisted longer in the soil in comparison to Metalaxyl-M [10]. Another investigation looked into the synergistic impacts of procymidone and FLU on the virulence and pesticide residue levels of cucumber *Botrytis cinerea*. The outcomes demonstrated a 59% and 86% reduction in residual levels on cucumber 21 d post-application when compared to the applications of procymidone and FLU individually, respectively [11]. Experts analyzed the control effectiveness and residues of prochloraz and FLU on tomato wilt. They observed significantly lower soil residue 50 days after compound application in comparison to the individual applications of each compound [12]. These findings support the results of the present study. Nevertheless, additional research is necessary to comprehend the reasons for the decrease in residual quantity after compounding versus when used independently. Pesticides are introduced into the soil through methods like spraying, root irrigation, and seed coating, where they undergo transformation and degradation. This process can significantly impact the soil microbiome, crucial for the soil ecosystem’s functioning. The soil microbiome is essential for converting soil materials, facilitating energy flow, and participating in biotransformation cycles, ultimately enhancing soil quality [13]. Evaluating the impact of pesticide pollution on the soil microbiome often involves assessing microorganism numbers, enzyme activity, and microbial community structure.

### 4.2. The Number of Soil Microbiota Is Changed by Pesticide

The effects of CAP and FLU on microbial activity under different treatment conditions were studied through the isolation and culturing of microbiomes using the dilution coating plate method. The abundance of various microbiota was monitored at different time points during the experiment. The results showed that treatments with CAP and FLU had varying inhibitory effects on fungal and bacterial colonies between 7 d and 35 d. However, after 35 days, colony numbers gradually increased and returned to control levels, suggesting an initial inhibition followed by the promotion of fungi and bacteria growth. Different pesticides can have specific impacts on the soil microbiome in terms of type and quantity. For example, tetraflurane has been shown to significantly suppress soil microbial biomass carbon and total bacterial populations, even at recommended field application rates [14]. This suppressive effect persists even after 90 days of cultivation. On the other hand, chlorpyrifos, salamand, and pretilachlor initially increase the microbial population in the soil before causing a decline, while carbendazim initially decreases the number of microbiota before eventually increasing it [15]. The number of bacteria and fungi in contaminated red soils and deep black soils increased significantly after the application of propiconazole compared to untreated soils [16]. Pesticide pollution has a dual impact on the number of microorganisms, leading to a decrease or disappearance of unsuitable microorganisms while also promoting the growth and accumulation of adaptive microorganisms [17]. Our findings confirm that pesticides can inhibit increases in the soil microbiota, although they may not always be directly toxic to them. In some cases, the microbiome can utilize pesticides as a source of carbon and nitrogen. This suggests that while pesticides can impact the diversity and abundance of the soil microbiome, their effects are complex and dependent on the type of pesticide and soil environment.

### 4.3. Soil Enzyme Activity Is Closely Linked to Soil Microorganism Populations

Soil enzymes are frequently utilized as indicators to evaluate the impact of pollutants on soil quality and biological activity, given their crucial role in energy flow and material circulation within the soil ecosystem [18]. Urease plays a crucial role in the breakdown of urea in soil [19]. Sucrase, a type of invertase, is impacted by various factors including soil organic matter content, chlorine and phosphorus levels, microbial population, and soil respiration intensity. The activity of invertase can serve as an indicator of soil biological activity, maturity, and fertility levels. Dehydrogenase, which is abundant in living microorganisms, is responsible for the decomposition of organic matter [20]. Various types of pesticides can have diverse effects on soil enzyme activities. The CAP treatment can decrease the activity of β-glucosidase in soil [6]. Fluoxastrobin has been shown to inhibit dehydrogenase and urease activities [21]. Conversely, propiconazole at recommended doses can enhance soil microorganism and enzyme activity [16]. This study delves into the impact of CAP and FLU seed treatment suspension agents on soil enzymes (urease, invertase, and dehydrogenase). The findings revealed that both CAP and FLU, either individually or in combination, had a transient inhibitory effect on urease and sucrase activities. However, the activity of dehydrogenase was stimulated by CAP alone or in conjunction with FLU. FLU initially inhibited dehydrogenase activity, but it later recovered during the experiment. Soil enzyme activity is closely linked to soil microorganism populations, with higher numbers of microbiota corresponding to increased enzyme activity [22]. The decrease in urease and invertase activities observed in this study may be due to a reduction in microbial populations (fungi and bacteria) in the soil. However, enzyme activity is restored when the microorganism population recovers. The increase in dehydrogenase activity with CAP application may be associated with its role in organic matter decomposition.

### 4.4. Mortierella Is Likely to Cause Pesticide Degradation

Soil microorganisms are vital for ecosystem sustainability and plant health. While pesticides can have negative effects on soil microbes, they can also act as a carbon source for specific groups of microbes. As microorganisms are key indicators of soil health, it is crucial to study the impact of pesticides on them [23]. In order to gain a deeper understanding of how various pesticide treatments affect soil microorganisms, an analysis was conducted on microbial biodiversity, community structure, composition, and function, as well as community networks [24]. Following the application of CAP and FLU to maize rhizosphere soil, the dominant fungal genera *Mortierella*, *Talaromyces*, and *Gibberella* were identified. *Mortierella* and *Gibberella* increased in relative abundance, while *Talaromyces* decreased. The research highlighted the important role of *Mortierella* in breaking down the herbicide diuron. *Mortierella’s* ability to degrade diuron was linked to its breakdown of other strains, with peak efficiency in carbon- and nitrogen-rich media [25]. A study identified *Mortierella* strains W8 and Cm1-45 as aerobic endosulfan-degrading fungi; these strains degraded over 70% and 50% of α- and β-endosulfan, respectively, in 28 days at 25 °C [26]. High-throughput sequencing revealed interactions among soil microbiota like *Vibrio*, *Fusarium*, *Gibberella*, *Meyerozyma*, *Exophiala*, and *Pseudoalteromonas*; the nitrate removal and denitrification rates reached optimal values of 100% and 44.27% under aerobic conditions [27]. The application of pesticides is known to lead to a decline in detrimental bacterial genera in the soil microbiome and an escalation in advantageous bacterial genera.

The FUNGuild prediction indicated that soil fungal communities were predominantly composed of undefined saprotroph and endophyte-litter saprotroph-soil saprotroph types. Following T2 and T3 treatments, there was a decrease in the relative abundance of saprophytic trophic fungi, potentially due to the saprophytic nature of *Talaromyces*, a member of the ascomycetes subphylum [28]. Conversely, after T1, T2, and T3 treatments, there was a relative increase in the abundance of endophyte-litter saprotroph-soil saprotroph-undefined saprotrophs, likely attributed to the rise in the relative abundance of *Mortierella*. Previous studies have identified *Mortierella* as an endophytic fungus present in tea leaves [29]. Endophytic fungi in plants exhibit developmental flexibility and perform diverse roles throughout different plant life cycles. Some fungi with intricate life histories possess multiple functional trophic types that can transition between each other, adapting to environmental changes [30]. Therefore, *Mortierella* may function as an endophytic fungus in healthy plant tissues and as a saprophytic fungus in aging or deceased tissues, contributing to organic degradation. These observations suggest that the increase in *Mortierella* abundance could aid in pesticide degradation. The study found that the relative abundance of norank_f__Vicianmibacteraceae and norank_f__norank_o__Vicianmibacterales significantly increased when CAP was used alone or in combination with FLU. Additionally, a separate study on pesticide residues in typical greenhouses in northern China revealed that Proteobacteria, Acidobacteria, and Bacteroidetes were the dominant phyla [31]. In the experiment, the residue of CAP in the soil decreased significantly after compounding, possibly due to changes in *Mortierella* and *Gibberella* caused by FLU. *Gibberella* might interact with other species in the soil microbiome to enhance deamination reactions, convert nitrate to nitrogen, and provide nutrients to Mortierella for pesticide degradation. Moreover, *Vicianmibacteraceae* and *Vicianmibacterales* could potentially contribute to the degradation of CAP.

## 5. Conclusions

This study utilized HPLC-MS/MS, colorimetric analysis, and Illumina Miseq sequencing to investigate the persistence of CAP and FLU in soil and their impact on the soil microbiome. When applied in combination, the levels of CAP and FLU residues decreased compared to individual applications, with CAP showing a significant reduction. The use of these compounds impacted both the enzyme activity and the abundance of soil microorganisms. This experiment demonstrated a clear relationship between the changes in enzyme activity and the number of microorganisms. The presence of these compounds, alone or together, influenced the abundance of fungi and bacteria near maize roots. Sequencing data analysis revealed that *Mortierella* played a vital role in breaking down CAP and FLU, while specific bacterial groups like *Vicianmibacteraceae* and *Vicianmibacterales* were identified as CAP degraders. Overall, the study found a significant decrease in CAP and FLU residues in soil, suggesting that their use could benefit the microbiome in maize rhizosphere soil. When used appropriately in agriculture, these chemicals are unlikely to cause pollution or leave residues in farmland.

## Data Availability

The data presented in this study are available in this article and Supplementary Materials.

## Supplementary Materials

The following supporting information can be downloaded at: https://www.mdpi.com/2039-4713/15/1/4/s1, Table S1: The nodes of co-occurring fungi networks obtained under CK, T1, T2 and T3; Table S2: The nodes of co-occurring bacterial networks obtained under CK, T1, T2 and T3; Figure S1: Soil microbial communities and the microbial functional diversity in soil (a) Fungal community structure analysis (b) FUNGuild function prediction of endophytic fungi (c) Bacterial community structure analysis.
