# Supplementary material for: Residual Dynamics of Chlorantraniliprole and Fludioxonil in Soil and Their Effects on the Microbiome
Source: J Xenobiot. 2024 Dec 30;15(1):4. doi: 10.3390/jox15010004 (PMC11840292; doi:10.3390/jox15010004)
Supplement: Supplementary file 1 [file jox-15-00004-s001.zip › jox-2948257-supplementary.pdf]

Table S1 The nodes of co-occurring fungi networks obtained under CK, T1, T2 and T3

Treatments	CK	T1	T2	T3
Ascomycota	120	122	121	118
Rozellomycota	2	2	3	2
Basidiomycota	14	18	16	15
unclassified Fungi	12	8	14	13
Mortierellomycota	11	12	16	14
Chytridiomycota	5	7	5	5
Glomeromycota	3	1	2	4
Blastocladiomycota	0	1	1	2
Total nodes	167	171	178	173

Table S2 The nodes of co-occurring bacterial networks obtained under CK, T1, T2 and T3

Treatments	CK	T1	T2	T3
Proteobacteria	102	100	96	110
Actinobacteriota	56	48	59	54
Acidobacteriota	18	41	22	36
Chloroflexi	61	34	59	27
Nitrospirota	0	4	5	4
Firmicutes	4	7	4	7
Gemmatimonadota	5	5	5	6
Cyanobacteria	11	4	7	5
Total nodes	257	243	257	249

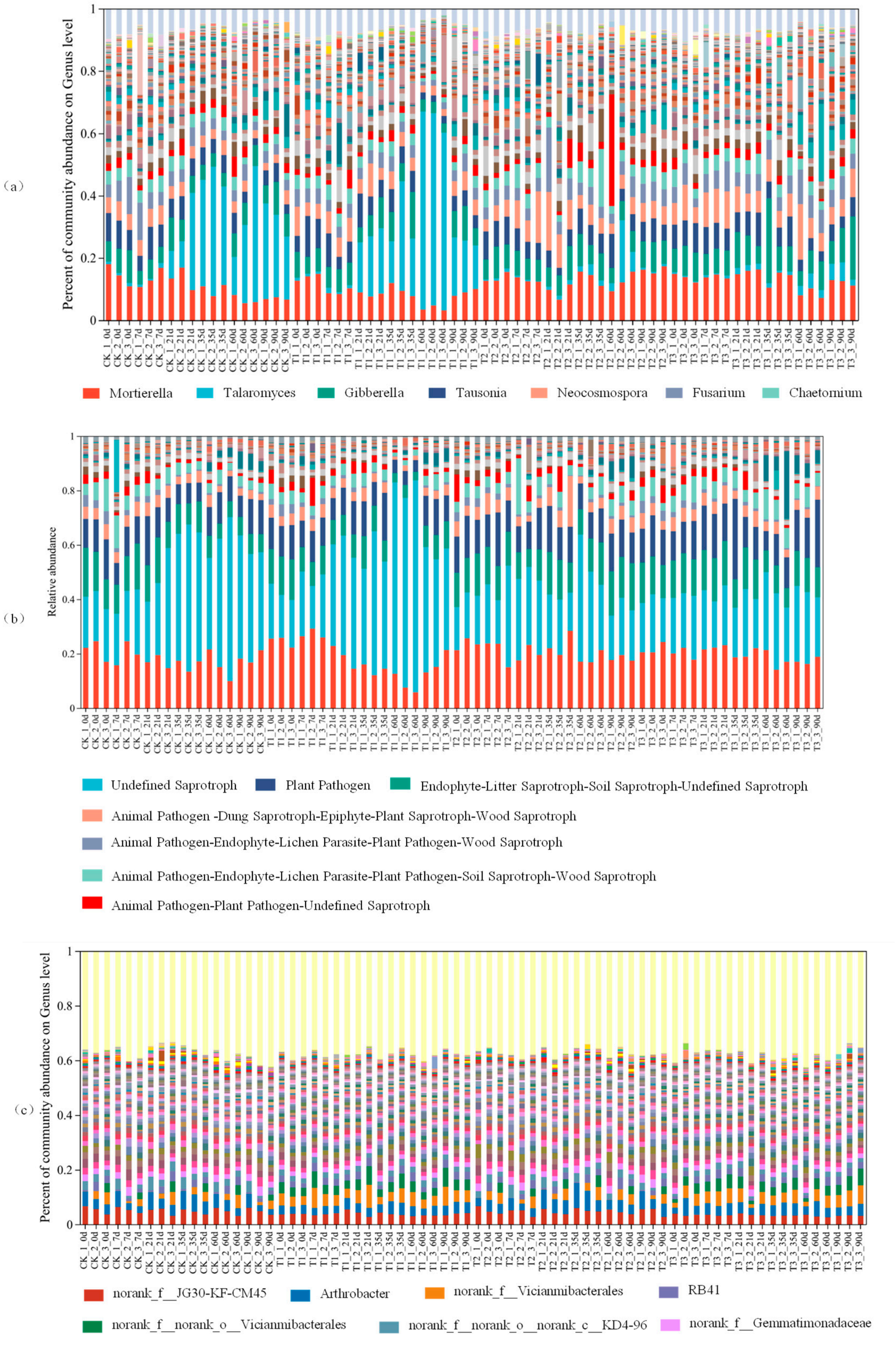

Figure S1. Soil microbial communities and the microbial functional diversity in soil (a) Fungal community structure analysis (b) FUNGuild function prediction of endophytic fungi (c) Bacterial community structure analysis
